# Evaluation of Knowledge and Practice of Resident Dentists in Iasi, Romania in the Management of Traumatic Dental Injuries: A Cross-Sectional Study

**DOI:** 10.3390/healthcare11091348

**Published:** 2023-05-08

**Authors:** Alice Murariu, Elena-Raluca Baciu, Livia Bobu, Simona Stoleriu, Roxana-Ionela Vasluianu, Monica Silvia Tatarciuc, Diana Diaconu-Popa, Petruța Huțanu, Gabriela Luminița Gelețu

**Affiliations:** 1 Department of Surgicals, Faculty of Dental Medicine, “Grigore T. Popa” University of Medicine and Pharmacy, 700115 Iasi, Romania; 2 Department of Implantology, Removable Prostheses, Dental Technology, Faculty of Dental Medicine, “Grigore T. Popa” University of Medicine and Pharmacy, 700115 Iasi, Romania; 3 Department of Cariology, Faculty of Dental Medicine, “Grigore T. Popa” University of Medicine and Pharmacy, 700115 Iasi, Romania

**Keywords:** traumatic dental injuries, dental avulsion, residents, knowledge, dental treatment

## Abstract

Dentists play an essential role in the treatment of dental and periodontal traumatic injuries by providing early and correct treatment. The purpose of the present study was to assess the level of knowledge of dental residents regarding dental trauma. The cross-sectional study was conducted at the Faculty of Dental Medicine within the “Grigore T. Popa” University of Medicine and Pharmacy in Iasi, Romania on a sample of 366 residents in General Dentistry, Pediatric Dentistry and Periodontology. To assess their knowledge, a questionnaire was created containing 18 questions about the clinical signs and therapy of dental trauma, with a focus on tooth avulsion. A very low level of knowledge (<25%) was found only for the type of splinting required to maintain an avulsed tooth on the arch. The highest number of correct answers was provided by the residents in Periodontology. Physiological serum as storage medium was recognized by a percentage of 75.9–80% of the subjects, and 60–77% of them recognized pulp necrosis as complication of dental avulsion. The study underlines the need to introduce in the curriculum of all categories of residents additional information, not only in the already existent theoretical form, but also as possible scenarios of various clinical situations.

## 1. Introduction

Traumatic Dental Injuries (TDIs) are injuries that occur after a traumatic impact on the tooth, the marginal periodontium, the oral mucosa and the alveolar bone [1].

Dental-periodontal traumas are among the most common oro-facial injuries, more frequently produced in childhood and adolescence, when they are associated with games and sports specific to the age period. For adults, sports activities, road accidents, and aggression are also mentioned [2].

According to the World Health Organization (WHO) modified classification (2018) [3], TDIs are grouped into:

- injuries to the hard-dental tissues and the pulp, which vary from enamel infraction to complicated crown-root fracture and root fracture;
- injuries to the periodontal tissues: concussion, subluxation, luxation and avulsion;
- injuries to the supporting bone;
- injuries to gingiva or oral mucosa.

Among these, dental avulsion is the most serious form of traumatic periodontal injury, defined as” complete separation of the tooth from the alveolar socket due to trauma” [4].

The prevalence of oral trauma in the world is quite variable, from 6% to 59%, according to the review conducted by Lam in 2016 [1]. Andersson found a 20% prevalence of dental trauma in children and adolescents in countries such as the United Kingdom and Scandinavia; in the United States 1 out of 4 adults presented trauma to the incisors, and in Canada, the prevalence of this type of injury was 15.5% in the 18–50 age group [5]. In Turkey, Sandalli reported a percentage of 38% for dental luxation and 20% for maxillary dental fractures in children aged 7–10 years [6]; avulsion of permanent teeth occured in a percentage of 0.5–16% of the dental injuries [7]. A Lithuanian study conducted between 2010 and 2016 showed that lateral luxation and intrusion occurred more often in primary dentition than in permanent dentition, while enamel-dentine fractures occurred more frequently in permanent than in primary dentition [8].

In Romania, Kovacs et al. [9] found a 24.5% prevalence of dental trauma in children and adolescents aged 1–18 years.

The differences observed in the prevalence of traumatic injuries may be due to geographical, cultural and economic differences. The prevalence of dental trauma was found to be higher in America compared to Asia or Europe [10,11].

The International Association of Dental Traumatology (IADT) has developed a comprehensive guide in four sections containing information and recommendations for the treatment of traumatic dental injuries in children and adults, useful for the emergency management of these lesions [12,13,14,15]. The guide, also translated into Romanian, helps dentists in making decisions and providing the best care, given the fact that a tooth with traumatic injuries is a dental emergency, the correct management of which allows to keep the tooth in the arch and avoid complications.

Complications following traumatic injuries to primary and permanent teeth may be represented by tooth discoloration, mobility, malocclusions, root and/or bone resorption, pulp necrosis with infection, and ultimately tooth loss. The consequences of such an impact can be dramatic, of a physical, emotional, economic and social nature [16].

The dentist has a primary role in providing emergency treatment by approaching a complex therapeutic attitude that includes procedures of dental-alveolar surgery, endodontics, pedodontics, periodontology and dental prosthetics. For this reason, dentists’ knowledge regarding the management of TDIs is essential. Early and correct diagnosis and emergency treatment can prevent functional, aesthetic and psychological problems [17].

Unfortunately, barriers exist in providing emergency treatment, which depend not only on doctors, but also on patients, in terms of insufficient knowledge and precarious financial situation [18].

In addition to raising public awareness, for example through media campaigns, health professionals, carers and teachers should receive information on how to deal with these unexpected serious injuries [5].

Although many studies exist in specialized literature, investigating the knowledge of dentists on TDIs [19,20,21,22,23,24,25,26,27,28,29,30], no study of this kind has been carried out in Romania so far.

The first information about the clinical signs and therapy of TDIs are presented to the students of the Faculty of Dental Medicine in Iasi, Romania in the 5th year, in the discipline of Oral and Maxillofacial Surgery. Afterwards, residency training involves courses and practical trainings, differentiated in terms of number and curriculum, depending on the specialty. These are more numerous in case of Oral Surgery residency training, and fewer for the specialty of Pediatric Dentistry (PD), which includes 30 h of course and practical training, General Dentistry (GD), with 2 h of course, without practical training and Periodontology (PERIO)—2 h of course, without practical training.

The purpose of the present study was to investigate the level of knowledge of resident dentists in the specialties of General Dentistry, Pediatric Dentistry and Periodontology from the Faculty of Dental Medicine in Iasi, Romania about the management of TDIs. The study was carried out after the completion of theoretical and practical training in this field by all three categories of resident dentists. The null hypothesis was that no differences in knowledge exist between the three categories of residents.

## 2. Materials and Methods

### 2.1. Research Design

A cross-sectional study was conducted between June 2022 and January 2023, in the Faculty of Dental Medicine in Iasi, on a sample of 366 resident dentists in years 1, 2 and 3 of training in GD, PD and PERIO specialties.

To conduct this study, the approval (No. 201/12.06.2022) of the ethics committee of “Grigore T. Popa” University of Medicine and Pharmacy of Iasi was obtained, as well as the informed consent of the participants to the study. Their anonymity was ensured, and they were offered the possibility to refuse participation to the study.

### 2.2. The Study Group

A convenience sampling method was used in this study. Initially, 416 residents were invited to participate in the study, of which 402 answered the request; later, 36 questionnaires were eliminated because they were incomplete, resulting in a number of 366 participants with complete questionnaires, and a study participation rate of 87.9%.

### 2.3. The Study Instrument

An original questionnaire was developed after literature research, adequate for the purpose of the study, namely assessment of knowledge about the management of TDIs, with an emphasis on dental avulsions [4,24,25,31].

The questions were adapted in accordance with the official recommendations of the guide developed by the IADT regarding the management of teeth with TDIs: coronal fractures, luxations and dental avulsions [13,14].

Several mandatory stages were completed for the questionnaire development. The first stage consisted of the translation from English into Romanian by 2 linguistic specialists, followed by the re-translation of the material back into English.

The final version contained 18 questions that were tested for internal consistency. Before starting the main study, a pilot study was carried out on a small group of participants (35 residents), with the aim of checking aspects related to the level of understanding and coherence of the questions. Finally, after some small necessary adjustments, the final form of the questionnaire was created, requiring a completion time of 7–10 min.

The questionnaire contains 4 sections. The first section is intended for general information of the residents participating in the study: age, gender, specialization.

The second section assesses knowledge of the management of traumatized teeth (Q1, Q2), the clinical aspects and the treatment of dental coronal and periodontal traumas (Q3, Q4, Q5, Q6).

The third section includes a number of 10 questions that focus on residents’ knowledge regarding the management of avulsed teeth including the possibilities of replantation of deciduous/permanent teeth (Q7 and Q8), tooth reinsertion into the socket (Q9), cleaning of the avulsed tooth (Q10), storage medium (Q11), time to arrive at the dental office (Q12), retention of the avulsed tooth—type of splinting and wearing period (Q13 and Q14), endodontic treatment (Q15), and possible complications that may occur (Q16).

The last 2 questions of the questionnaire, in the fourth section, evaluate the residents’ perception of the knowledge they possess in this field (Q17), as well as their desire to participate in additional courses (Q18).

To classify the knowledge level (KL), the following criteria were used [19]:

- excellent, when the percentage of correct answers ranged from 100% to 90%;
- good, when the percentage of correct answers ranged from 90% to 70%;
- moderate, when the percentage of correct answers ranged from 70% to 50%;
- low, when the percentage of correct answers ranged from 50% to 25%;
- very low, when the percentage of correct answers was lower than 25%.

### 2.4. Data Analysis

Statistical analysis was performed using the Statistical Package for Social Sciences program (SPSS Inc., Chicago, IL, USA, version 20 for Windows). Descriptive data were analyzed using frequency and percentage and crosstabulation, and the Chi-square test was used to identify differences between groups, with a significance level of 5% (*p* < 0.05).

## 3. Results

### 3.1. Study Participants

Out of the 366 participants, 138 (37.7%) were residents in GD, 120 (32.8%) were residents in PD, and 108 (29.5%) were residents in PERIO. The gender distribution in the study sample was 150 males (40.9%) and 216 females (59.1%). The age range of study participants was 25–42 years, with a mean of 30.01 ± 0.650 years, and the following distribution on age groups: 261 (71.3%) in the 25–30 years group, 70 (19.1%) in the 31–35 years group, and 35 (9.6%) in the 36–42 years group.

The characteristics of the study participants are presented in Table 1.

### 3.2. Knowledge of the Clinical Aspects and the Management of Injuries to the Hard Dental Tissues and Periodontal Tissues

The answers to the questions that refer to residents’ knowledge regarding the clinical signs and therapy of TDIs lesions are presented in Table 2.

The most subjects who met patients with traumatic dental-periodontal injuries in their current practice (Q1) were the residents in GD (74%), followed by the residents in PERIO (63%) and in PD (61.7%).

Question Q2 refers to the management of the situation of witnessing to the occurrence of a dental-periodontal trauma. The correct answer (personal travel to the nearest medical facility with the injured person) was selected to the greatest extent by the residents in PD (60%), (*p* = 0.022).

The correct answer to question Q3 (What is the predominant clinical sign of acomplicated crown fracture?), pulp hemorrhage, was offered by 55% of PD residents, and only 25.9% of PERIO residents (*p* = 0.001). However, tooth mobility as the predominant clinical sign of a dental luxation (Q4), was known by the majority of residents.

The percentage of subjects who gave the correct answer to question Q5, referring to the emergency treatment of a complicated crown fracture in a permanent tooth (pulp excision) was the highest among PD residents (60%), followed by PERIO residents (48.2%), and GD residents (46.4%) (*p* = 0.043).

The highest percentage of correct answers to question Q6 What is the emergency treatment for a luxated permanent tooth), was found among PERIO residents (64.8%), (*p* = 0.014).

The level of knowledge for this section of the questionnaire was classified as follows:

- for GD residents: good for Q4 and low for Q2, Q3, Q5 and Q6;
- for PD residents: good for Q4, moderate for Q2, Q3 and Q5, and low for Q6;
- for PERIO residents: good for Q4, moderate for Q2 and Q6, and low for Q3 and Q5.

### 3.3. Knowledge of the Clinical Management of Dental Avulsion, and Continuing Education

Section 3 of the questionnaire refers to the management of avulsed teeth. The answers are presented in Table 3.

The first two questions of this section refer to the possibility of replantation of deciduous (Q7) and permanent teeth (Q8). The correct answer to question Q7 (No) was given by most PERIO residents (79.6%), followed by GD residents (50.7%) and PD residents (45%), with significant differences between groups (*p* = 0.001).

For question Q8, the level of knowledge was higher than for the previous question, with the correct answer (Yes) given by the majority of residents, more than 85%.

A large percentage of participants also gave the correct answer to question Q9, regarding the reinsertion of the avulsed tooth into the socket: 85.2% of the PERIO residents, 81.7% of the PD residents, and 76.8% of the GD residents.

The correct answer to question Q10, referring to the cleaning of the avulsed tooth, (Wash with physiological serum) was offered to the greatest extent by the GD residents (89.9%). Significant differences were found between groups (*p* = 0.011).

For question Q12 (What is the optimal time to arrive to the dental office?), the correct option (30 min) was selected to the greatest extent by PERIO residents (85.1%), with significant differences between groups (*p* = 0.009).

Questions Q13 and Q14 evaluate the level of knowledge regarding the type of splinting (Q13) and the retention period (Q14). Correct answers regarding the type of splinting (flexible), were given by a small number of residents, only 8.3% for the PD group, and the 10–14-day retention period was correctly identified by only 28.3% of PD residents.

Another question to which the percentage of correct answers varied was Q15 (When should endodontic treatment be performed?). From the three groups of residents, only 63.4% of the PD group marked the correct answer (7–10 days after replantation). A percentage of 38.9% of PERIO residents considered that endodontic treatment should be carried out after the patient complains of symptoms. Significant differences were found between groups (*p* = 0.046).

The percentage of residents who consider themselves satisfied with the knowledge they had (Q17) varied from 51.9% for the PERIO group to 43.3% for the PD group. Most of the interviewees stated that they would be interested in acquiring additional knowledge on practical aspects of TDIs (Q18) (significant differences, *p* = 0.001).

The level of knowledge for the second section of the questionnaire was classified as follows:

- very low for question Q13 for all three categories of residents;
- for GD residents: good for questions Q8, Q9 and Q10, moderate for Q7 and Q12, and low for questions Q14 and Q15;
- for PD residents: good for questions Q8, Q9 and Q10, moderate for Q12, Q15, and low for Q7, Q13, Q14;
- for PERIO residents: good for Q7, Q8, Q9, Q10, Q12, and low for questions Q13, Q14 and Q15.

**Figure 1:**
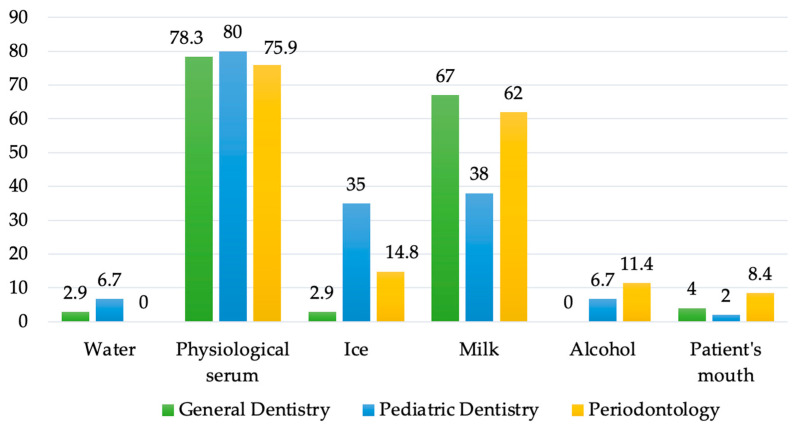
Distribution of answers to question Q11 (What is the recommended storage medium for an avulsed tooth?).

Figure 1 shows the residents’ answers to question Q11 (What is the recommended storage medium for an avulsed tooth?). It is observed that the correct storage medium, physiological serum, was identified by most of the residents: 80% of the PD group, 78.3% of the GD group, and 75.9% of the PERIO group. The level of knowledge was considered to be good.

The second correct option, milk, was identified by a smaller percentage of participants, resulting a moderate level of knowledge for GD and PERIO groups and low level for PD group.

**Figure 2:**
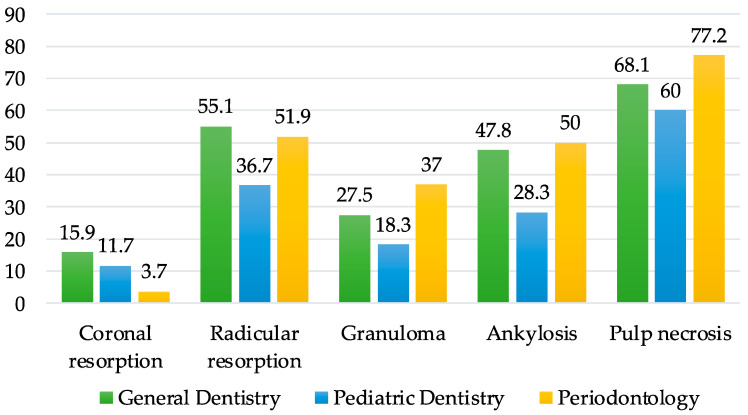
Distribution of answers to question Q16 (What are the possible complications after tooth replantation?).

Figure 2 presents subjects’ answers to question Q16 (What are the possible complications after tooth replantation?). The correct answers were given by a percentage of residents that varies from 68.1% to 77.2% for pulp necrosis (moderate level), from 36.7% to 55.1% for radicular resorption (moderate level for GD and PERIO, low level for PD), and from 28.3% to 50% for ankylosis (moderate level for PERIO, low level for GD and PD).

## 4. Discussion

The present research included the most relevant questions regarding the clinical picture and the emergency treatment of dental-periodontal traumatic injuries, so as to allow an overview of the knowledge of resident doctors from the three different fields of dental practice: General dentistry, Pediatric Dentistry and Periodontology, which have training hours for this subject in their curricula.

Among all the residents, those who encountered traumatic dental injuries in the current practice in the highest percentage were those from the GD specialty (74%), possibly due to the fact that they carry out their activity in private dental offices where they provide specialized treatments to adult patients.

The first six questions of the questionnaire focus on the elementary notions regarding crown fractures and dental luxations.

Regarding the knowledge related to traumatic crown lesions and their treatment, the percentage of correct answers varied from 25.9% to 55%, and from 46.4% to 60%, respectively.

Among the three categories of residents, those from the GD have the least knowledge (low level), although they met patients with TDIs in the highest percentage.

On the other hand, the level of knowledge regarding traumatic injuries such as dental luxations was good in what concerns the predominant clinical sign (tooth mobility), but lower regarding the treatment. Differences were found depending on the specialization: the residents from the PERIO specialty had the most correct answers (64.8%—moderate level), while the PD residents had the fewest correct answers (low level).

In a similar research study, in Turkey, Cinar et al. [32] found a higher percentage of correct answers regarding the clinical signs of crown fractures (60.8%) compared to our study, but lower for dental luxations (57%). In Australia, Jadav et al. [33] found a lower percentage of correct answers regarding the treatment of choice for crown fractures (pulpectomy—48%). In India, in a study that included a group of dentists specializing in GD, Ravikumar et al. [34] found a higher percentage of correct answers (65%) concerning crown fractures with pulp exposure, compared to the present study, and in Brazil, the reported percentage was even higher (75.3%) [35].

The second part of the questionnaire evaluated the knowledge related to dental avulsion. According to the guide provided by the IADT, the deciduous avulsed tooth should not be replanted [14]. The answers to question Q7 demonstrated that almost one third of the residents included in the present study did not know this.

In a study conducted in the United Arab Emirates, Alyasi et al. [21] found a much higher percentage of dentists (74%) who considered that the deciduous tooth can be replanted, while in another study conducted in Croatia, the percentage of incorrect answers of pediatricians to this question was 59.7% [25].

Before being transported, the avulsed tooth must be washed with physiological serum (Q10); correct answers to this question were given by 75–88.4% of the residents participating in the study, a percentage similar to that given by dentists in Turkey (80%) [36].

The ideal treatment of tooth avulsion that can ensure long-term success is immediate replantation. Transport media are used to maintain the viability of periodontal ligament cells, increase their survival, and prevent any damage that could cause future tooth loss, such as ankylosis and resorption [37,38,39].

The correct storage medium (physiological serum) was recognized by 75.9–80% of the investigated residents; for milk, the percentage of correct answers was lower (38–62%). Among the three groups of residents, the percentage was the lowest for residents from the PD specialty. Also, this same category of residents, in a percentage of 35%, considered that ice can be used as a storage medium, which is an incorrect answer. This aspect is surprising, because the training curriculum in this specialty includes, in addition to theoretical information, hours of practical training, unlike the other two specialties, where there is only one theoretical course. The only explanation would be that they have not yet encountered such traumatic injuries in their current practice.

In the specialized literature, the percentage of correct answers regarding physiological serum and milk as the storage medium varies from 100% for milk in the United Arab Emirates [21], to 74.7% among Italian dentists [24], 15.8% correct answers for dentists in China [27], and 11.2% in Germany [38].

A low level of knowledge was observed in the present study regarding the type of splinting and its retention period. The IADT guidelines suggest wearing a flexible splinting for two weeks to decrease the risk of ankylosis and starting endodontic treatment 7–10 days after replantation of an avulsed permanent tooth [14].

The present research shows that a small percentage of participants recognized the correct type of splinting recommended (the flexible one), which places the level of knowledge in the very low category, and the correct answers to question Q14 How long should the splinting be worn?) place the level of knowledge in the low category.

This percentage of correct answers is lower than that provided by dentists in Italy, where 33.2% of them correctly identified the type of flexible splinting, or in China, where the percentage was even higher (45.1%) [24,27].

In tooth replantation, replacement resorption was observed in 94.12% of cases, and the time the tooth stayed in the extra-alveolar environment and the storage medium of the avulsed tooth are very important elements for the repair of the periodontal ligament [40].

The correct answers regarding the most common complications that occur after the replantation of an avulsed tooth are pulp necrosis, ankylosis, and root resorption.

For pulp necrosis, the level of knowledge was moderate, and for ankylosis, the level varied between moderate and low; the same level was also found for radicular resorption. Among the three categories of residents, those from the PD specialty had the lowest level of knowledge, with the lowest percentage of correct answers.

In India, a study on 514 dentists with various specialties showed that endodontists and pedodontists had the highest level of knowledge concerning the complications after tooth replantation [41].

Regarding the knowledge held, almost half of the participants admitted that they were not satisfied with the knowledge they had about TDIs, but participation in additional courses was only agreed by 60% of the PD residents, while the GD and PERIO residents agreed in a percentage of more than 80%.

Such information can be provided starting with the faculty, when students can access other educational methods based on “problem-based learning, to effectively prevent dental trauma complications” [42].

The importance of the topic is explained by the speed of applying a correct treatment in the case of a TDIs, which requires the dentist to have complete and varied knowledge about the management of patients with such injuries. From the answers given, it can be seen that the basic information presented in the faculty is not enough, so the residency training curriculum is essential.

The present study has a number of limitations: first, the questionnaire was focused on dental avulsion and less on other traumatic dental-periodontal injuries, for which only a few relevant questions were chosen. The second limitation refers to the fact that the study included only residents from three specializations, which have hours dedicated to dental traumatology in their training curriculum and, does not reflect the knowledge of the other categories of residents. In the future, the research can be extended to categories of residents that were not the subject of the current study (Endodontics, Orthodontics and Dental Prosthetics).

Following the statistical analyses, the null hypothesis was rejected and thus it was established that differences exist between the knowledge of the three categories of residents.

## 5. Conclusions

The conclusions of the present study are as follows:

- The results of the study demonstrate that the level of knowledge of the resident dentists in the management of TDIs is not very high: none of them identified the correct answers at a level of 90%.
- Among the three categories included in the study, the most correct answers were given by the residents from the Periodontology specialty.
- The study underlines the need to introduce additional information in the curriculum of all categories of residents, such as different clinical scenarios, the importance of the radiological examination in the management of dental trauma, and root treatment techniques in complicated crown and root fractures.

## Figures and Tables

**Table 1:** Characteristics of study participants (N = 366).

Variables		N	%
Participants	General Dentistry (GD)	138	37.7
	Pediatric Dentistry (PD)	120	32.8
	Periodontology (PERIO)	108	29.5
Gender	Male	150	40.9
	Female	216	59.1
Age (mean ± SD ^1^)	25–30 years	261	71.3
	31–35 years	70	19.1
	36–42 years	35	9.6
	30.01 ± 0.650		

^1^ SD-standard deviation.

**Table 2:** Assessment of the knowledge about the clinical aspects and the management of TDIs (The correct answers are marked in italics).

	General Dentistry (GD) N (%) (KL)	Pediatric Dentistry (PD) N (%) (KL)	Periodontology (PERIO) N (%) (KL)	*p*-Value
Q1. Have you met patients with TDIs in current practice?				
Yes	102 (74)	74 (61.7)	68 (63)	0.267
No	36 (26)	46 (38.3)	40 (37)	
Q2. What do you do if you witness an accident in which TDIs occur?				
I calm down the injured person and provide the necessary information	52 (37.7)	42 (35)	50 (46.3)	0.022 *
*I go to the nearest medical facility with the injured person*	*66 (47.8)* *(low)*	*72 (60)* *(moderate)*	*58 (53.7)* *(moderate)*	
I do not know what to do	20 (14.5)	6 (5)	0	
Q3. What is the predominant clinical sign of a complicated crown fracture?				
*Pulp hemorrhage*	*60 (43.5)* *(low)*	*66 (55)* *(moderate)*	*28 (25.9)* *(low)*	0.001 *
Sensitivity to stimuli	54 (39.1)	42 (35)	78 (72.2)	
Tooth mobility	20 (14.5)	10 (8.3)	2 (1.9)	
I do not know	4 (2.9)	2 (1.7)	0	
Q4. What is the predominant clinical sign of a dental luxation?				
Pulp hemorrhage	0	4 (3.3)	4 (3.7)	0.132
Pain to chemical stimuli	2 (1.4)	14 (11.7)	6 (5.6)	
*Tooth mobility*	*132 (95.7)* *(good)*	*96 (80)* *(good)*	*96 (88.9)* *(good)*	
I do not know	4 (2.9)	6 (5)	2 (1.8)	
Q5. What is the emergency treatment of a complicated crown fracture in a permanent tooth?				
*Pulp excision*	*64 (46.4)* *(low)*	*72 (60)* *(moderate)*	*52 (48.2)* *(low)*	0.043 *
Partial pulpotomy	50 (36.2)	38 (31.6)	48 (44.4)	
Tooth repositioning	24 (17.4)	10 (8.4)	8 (7.4)	
Q6. What is the emergency treatment for a luxated permanent tooth?				
Tooth immobilization for 2 weeks	60 (43.5)	68 (56.6)	32 (29.6)	0.014 *
Tooth repositioning	12 (8.7)	14 (11.7)	6 (5.6)	
*All previous statements*	*66 (47.8)* *(low)*	*38 (31.7)* *(low)*	*70 (64.8)* *(moderate)*	

Note: Chi square test. * Significance level of 0.05; KL = knowledge level.

**Table 3:** Assessment of dental avulsion knowledge and continuing education among participants. (The correct answers are marked in italics).

	General Dentistry (GD) N (%) (KL)	Pediatric Dentistry (PD) N (%) (KL)	Periodontology (PERIO) N (%) (KL)	*p*-Value
Q7. Do you think deciduous teeth can be replanted?				
Yes	52 (37.7)	44 (36.7)	22 (20.4)	0.001 *
*No*	*70 (50.7)* *(moderate)*	*54 (45)* *(low)*	*86 (79.6)* *(good)*	
I do not know	16 (11.6)	22 (18.3)	0	
Q8. Do you think permanent teeth can be replanted?				
*Yes*	*128 (92.8)* *(good)*	*102 (85)* *(good)*	*106 (98.1)* *(good)*	0.090
No	8 (5.8)	10 (8.3)	2 (1.9)	
I do not know	2 (1.4)	8 (6.7)	0	
Q9. If you witness a tooth avulsion, would you try to reinsert the tooth into the socket?				
*Yes*	*106 (76.8)* *(good)*	*98 (81.7)* *(good)*	*92 (85.2)* *(good)*	0.410
No	26 (18.8)	14 (11.7)	8 (7.4)	
I do not know	6 (4.3)	8 (6.7)	8 (7.4)	
Q10. If the tooth is dirty, how do you clean it?				
Wash with water	12 (8.7)	30 (25)	16 (14.8)	0.011 *
*Wash with physiological serum*	*124 (89.9)* *(good)*	*90 (75)* *(good)*	*86 (79.6)* *(good)*	
Rub with toothbrush	2 (1.4)	0	0	
Keep in fluoride mouthwash for 10 min	0	0	6 (5.6)	
Q12. What is the optimal time to arrive to the dental office?				
*30 min*	*94 (68.1)* *(moderate)*	*68 (56.7)* *(moderate)*	*92 (85.1)* *(good)*	0.009 *
Several hours	32 (23.3)	38 (31.7)	14 (13)	
The first 24 h	10 (7.2)	4 (3.3)	2 (1.9)	
I do not know	2 (1.4)	10 (8.3)	0	
Q13. What type of splinting is used for avulsed teeth?				
Rigid type	104 (75.4)	86 (71.7)	90 (83.3)	0.204
*Flexible type*	*22 (15.9)* *(very low)*	*10 (8.3)* *(very low)*	*10 (9.3)* *(very low)*	
Splinting is not necessary	4 (2.9)	4 (3.3)	0	
I do not know	8 (5.8)	20 (16.7)	8 (7.4)	
Q14. How long should the splinting be worn?				
*10–14 days*	*62 (44.9)* *(low)*	*34 (28.3)* *(low)*	*44 (40.7)* *(low)*	0.090
28–30 days	46 (33.3)	36 (30)	34 (31.5)	
60 days	22 (16)	24 (20)	10 (9.3)	
I do not know	8 (5.8)	26 (21.7)	20 (18.5)	
Q15. When should endodontic treatment be performed?				
Immediately after replantation	8 (5.8)	6 (5)	4 (3.7)	0.046 *
*7–10 days after replantation*	*62 (45)* *(low)*	*76 (63.4)* *(moderate)*	*34 (31.5)* *(low)*	
30 days after replantation	22 (15.9)	10 (8.3)	28 (25.9)	
When the patient has symptoms	44 (31.9)	28 (23.3)	42 (38.9)	
I do not know	2 (1.4)	0	0	
Q17. Do you consider yourself satisfied with the knowledge you have?				
Yes	68 (49.3)	52 (43.3)	56 (51.9)	0.640
No	70 (50.7)	68 (56.7)	52 (48.1)	
Q18. Would you like additional information about TDIs management?				
Yes	128 (92.8)	72 (60)	90 (83.3)	0.001 *
No	10 (7.2)	48 (40)	18 (16.7)	

Note: Chi square test. * Significance level of 0.05; KL = knowledge level.

## Data Availability

The data that support the findings of this study are available on request from the corresponding author.
